# Pilot Scale Production of a F_420_ Precursor Under Microaerobic Conditions

**DOI:** 10.1002/biot.70002

**Published:** 2025-03-18

**Authors:** Annika Lenić, Bettina Bardl, Florian Kloss, Gundela Peschel, Ivan Schlembach, Gerald Lackner, Lars Regestein, Miriam A. Rosenbaum

**Affiliations:** ^1^ Bio Pilot Plant, Leibniz‐Institute for Natural Product Research and Infection Biology Hans‐Knöll‐Institute Jena Germany; ^2^ Faculty of Biological Sciences Friedrich‐Schiller‐University Jena Germany; ^3^ Transfer Group Anti‐infectives Leibniz‐Institute for Natural Product Research and Infection Biology Hans‐Knöll‐Institute Jena Germany; ^4^ Chair of Biochemistry of Microorganisms, Faculty of Life Science: Food, Nutrition and Health University Bayreuth Bayreuth Germany

**Keywords:** deazaflavin purification, F_O_, F_420_, *Mycobacteria*, process scale‐up, redox cofactor synthesis

## Abstract

The functional investigation of redox cofactors is important for many potential biocatalytic processes, yet limited access to these molecules is often hampering efficient research activities. Deazaflavin‐dependent enzymes mediate a range of biochemical redox reactions in prokaryotes. Coenzyme F_420_‐dependent enzymes are interesting for asymmetric redox biocatalysis and other challenging reactions, but low F_420_ titers harvested from natural producers and engineered host strains so far limit intensive investigation of these enzymes. F_O_ is a natural precursor of F_420_, which already shares many of the redox properties and was previously confirmed as a surrogate for F_420_ in certain enzymes. Here, we focused our research on the development of an overall process workflow from a 30‐L pilot scale stirred tank bioprocess to an optimized downstream purification process to obtain pure F_O_ from an engineered *Escherichia coli* host strain. We were able to shift the cofactor synthesis from riboflavin to F_O_ via the implementation of oxygen‐limited process conditions during heterologous *fbiC* expression and reached a final titer of 5.05 mg L^−1^ F_O_ in our fermentation broth, which for the first time allows the purification of relevant amounts for subsequent experiments. Online fluorescence measurement within the bioreactor system made it possible to monitor F_O_ formation and confirmed growth‐associated F_O_ biosynthesis.

## Graphical Abstract and Lay Summary

This work studies the influence of oxygen availability on heterologous biosynthesis of the redox co‐factor F_O_ with an engineered *E. coli* strain up to pilot scale. Microaerobic conditions led to a counter selection of the unwanted natural side product riboflavin and substantially increased titers of F_O_.

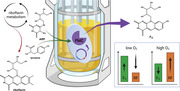

**Abbreviations**
AC: adsorption chromatography
A_R_
: gas‐liquid surface (m^2^)
CTR: carbon transfer rate (mmol L^−1 ^h^−1^)
DO: dissolved oxygen (%)
DOT: dissolved oxygen tension (%)
D_R_
: reactor diameter (m)
d_stirrer_
: stirrer diameter (m)
g: gravity constant (m s^−1^)
Ne: power number, unaerated (‐)
n_stirrer_
: stirring rate (s^−1^)
OTR_max,E_
: maximum OTR, derived from experiments (mmol L^−1 ^h^−1^)
OTR_max_
: maximum OTR, calculated (mmol L^−1 ^h^−1^)
OTR: oxygen transfer rate (mmol L^−1 ^h^−1^)
P_R,30L,aerated_
: absolute power input 30‐L reactor, aerated (W)
P_R,7/30L,unaerated_
: absolute power input unaerated (W)
P_R,7L,aerated_
: absolute power input 7‐L reactor, aerated (W)
q_g_
: aeration rate (m^3^s^−1^)
u_g_
: gas velocity (m s^−1^)
V_G_
: gas flow volume (L h^−1^)
V_L_
: culture volume (L)
V*_norm_*
: ideal gas volume (L mol^−1^)
V_R_
: filling volume (m^3^)
y_C_ *_O2,in_*
: concentration of carbon dioxide in gas in‐flow (‐)
y_C_ *_O2,out_*
: concentration of carbon dioxide in off‐gas (‐)
y*_O2,in_*
: concentration of oxygen in gas in‐flow (‐)
y*_O2,out_*
: concentration of oxygen in off‐gas (‐)

## 1 Introduction

Oxidoreductases and their corresponding redox cofactors are important for many bioprocesses and chemical transformations. Unfortunately, when non‐standard cofactors like deazaflavins are involved, research on these cofactor‐dependent oxidoreductases is often hampered by the limited availability of the latter. Deazaflavin‐dependent enzymes gained more interest within recent years, as their distribution among bacteria and archaea was found to be more widespread than originally expected [1, 2, 3, 4]. F_420_ belongs to the group of deazaflavin cofactors, and its distribution has been attributed to archaea and bacteria species [5]. Several studies have evaluated the relevance of F_420_‐dependent enzyme families for biocatalysis [6, 7, 8, 9, 10, 11, 12] or predicted a great potential for biotechnological use. However, as the family of F_420_‐dependent enzymes is highly diverse and the structure and function of a wide range of these enzymes are not characterized yet, the topic of F_420_‐dependent enzymes is still underexplored [11]. In that regard, these articles also highlighted the need for the development of improved preparative production of cofactors and research directed into cofactor recycling [11, 13].

Coenzyme F_420_ itself is an inspirational redox cofactor that mediates a variety of hybrid transfer reactions [14] and contributes, for example, to antibiotic biosynthesis [15] or stress response in actinobacteria [16] and methanogenesis in archaea [17, 18]. However, investigation of F_420_‐dependent enzymes is restricted by the limited availability of the compound. The best natural producers only provide low quantities of F_420_ or are difficult to cultivate [19, 20]. A metabolically engineered strain of *Mycobacterium smegmatis* produced about 10 times higher amounts of the cofactor than the wildtype but is not ideally suited for bioprocess upscaling due to cultivation conditions and safety concerns [21]. The first heterologous productions of the cofactor in *Escherichia coli* were achieved by several groups [2, 19] and optimized in recent follow‐up studies [22, 23, 24].

In this work, our research interest is in the F_420_‐derivative F_O_. F_O_ (7,8‐didemethyl‐8‐hydroxy‐5‐deazariboflavin) is a stable precursor during natural F_420_ biosynthesis and typically found in the culture supernatant of F_420_ producers [20, 23, 25, 26]. The precursor is mainly known as an antenna‐chromophore for DNA photolyases [27] that enhances the efficiency of DNA repair under low light conditions [28]. Interestingly, for some F_420_‐dependent enzymes, it was shown that F_O_ can substitute for F_420_ in catalytic reactions [29, 30, 31]. On the contrary, some enzymes need the phosphorylated version, namely F_O_P [6], or even the fully mature cofactor for their activity [32, 33, 34, 35]. However, while investigating F_O_P synthesis and enzymatic utilization in *Escherichia coli* and *Saccharomyces cerevisiae*, F_O_ substitution was necessary in *Saccharomyces*, as the strain was not able to perform the biosynthesis itself and could only further convert F_O_ to its phosphorylated form [36, 37]. Further, members of our team could recently show that F_O_ is involved in the maintenance of a bacterial‐fungal symbiosis, which hints towards a completely new role of F_O_ in interspecies communication [38]. Overall, not many studies actively investigate the effect of the different variants of F_420_‐derivatives on the activity of the investigated enzymes or reactions. To enable a more thorough investigation of possible F_O_‐dependent reactions and processes, we here focused on the bioproduction of F_O_ as our research goal.

The first synthetic chemistry approach for F_O_ synthesis was reported in 2015 [29]. This protocol later was simplified and modified regarding the reductive amination procedure, combining two previous steps into one and thereby increasing the efficiency of the process [6]. In contrast, biological synthesis approaches, so far, were only achieved on small scales and with low overall yield [25, 39].

The responsible enzyme for F_O_ biosynthesis, F_O_ synthase, is either a two‐domain fusion protein in many bacteria, encoded by *fbiC*, [2, 40] or consists of two polypeptides, typically encoded by *cofG* and *cofH* in archaea [39, 41]. F_O_ synthase utilizes 5‐amino‐6‐ribitylamino‐2,4(1*H*,3*H*)‐pyrimidinedion (ARP) and tyrosine as substrates for an oxidative coupling reaction [42]. The enzyme harbors two radical SAM domains and uses two separate 5’‐deoxyadenosyl radicals to catalyze F_O_ formation [42]. ARP also represents a precursor of the riboflavin synthesis pathway [43, 44].

**FIGURE 1:**
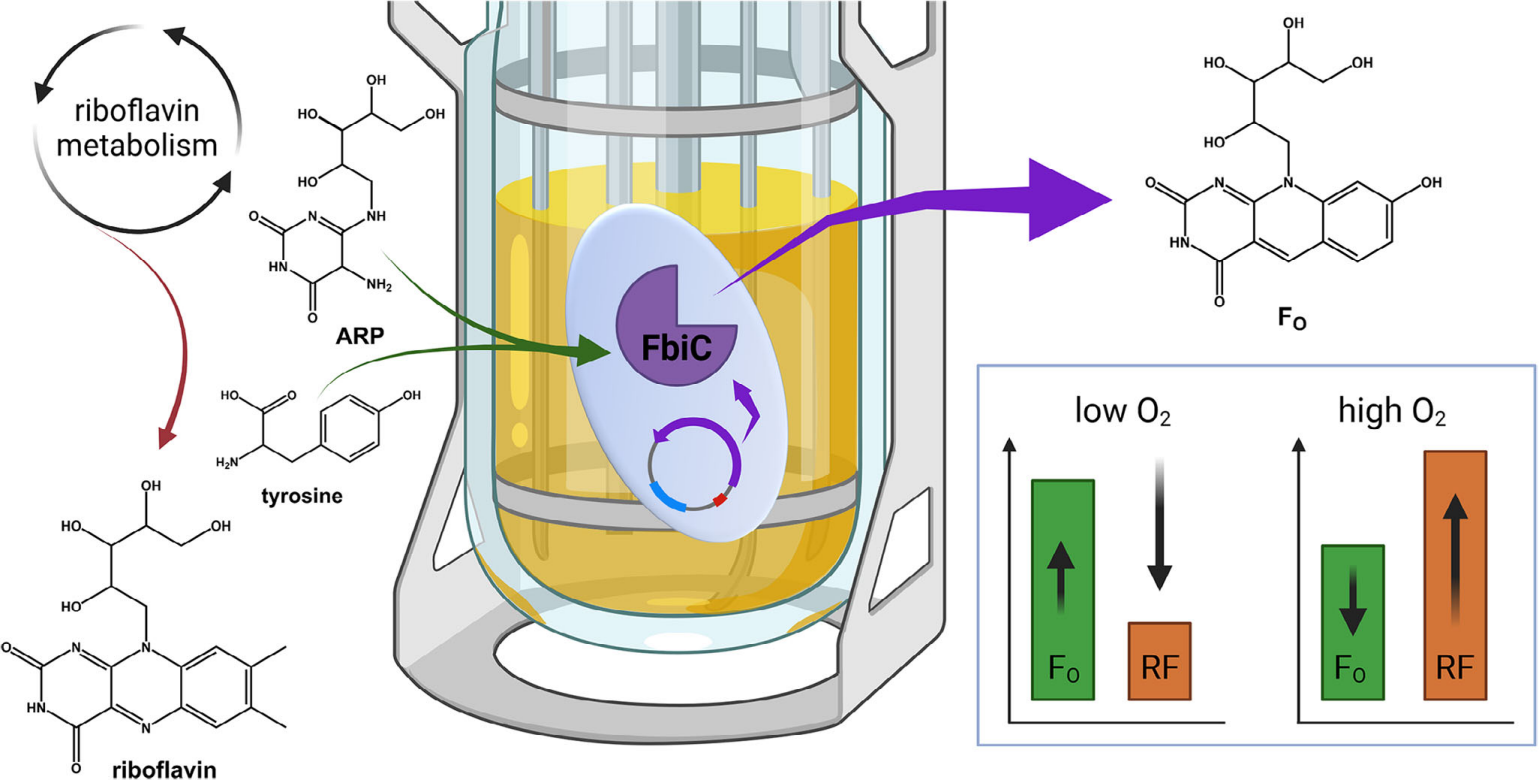
Biosynthesis of F_O_ in *E. coli* pDB048 through the homologous two‐domain FbiC F_O_‐Synthase from *Mycetohabitants rhizoxinica*, which utilizes 5‐amino‐6‐ribitylamino‐2,4(1H,3H)‐pyrimidinedion (ARP) and tyrosine as substrates. The availability of oxygen greatly affects the ratio of F_O_ and riboflavin (RF).

In this study, we installed and optimized the F_O_ bioprocess in *E. coli* via microaerophilic process conditions to achieve higher product titers and established a sufficient downstream purification process that allows for further investigation of F_420_ substitution through F_O_ in enzymatic reactions (Figure 1). During both the biosynthesis and the purification of F_O_, the processes had to be steered away from riboflavin as a ubiquitous cofactor. We performed a robust scale‐up of the process from shake flask into stainless steel bioreactors. The final working volume during this study was 20 L in 30‐L bioreactors.

The two‐domain F_O_ synthase (FbiC) from *Mycetohabitans rhizoxinica* [2] was expressed via an inducible plasmid system in the biotechnological host *E. coli* BL21 (Figure 1). We chose this enzyme for biosynthesis as it is derived from a Gram‐negative bacterium and might therefore perform more efficiently in *E. coli* than the well‐known archaeal or mycobacterial strains [36, 39].

Controlled oxygen conditions to steer carbon away from biomass formation and towards enhanced product formation were used in other studies to produce bulk chemicals and polymers in different host strains [45, 46, 47, 48]. Furthermore, a previous study showed that the availability of oxygen can be an important factor to enhance redox‐cofactor formation [49, 50]. The effect of high or low dissolved oxygen (DO) conditions on riboflavin synthesis and specifically, the upregulation of the Embden‐Meyerhof‐Pathway and the downregulation of the Pentose‐Phosphate‐Pathway under low DO conditions was extensively investigated in the common riboflavin production host *Bacillus subtilis* [51]. Another study examined the genetic effects of nitrogen metabolism and DO limitation on the regulation of riboflavin synthesis in *B. subtilis* [52]. However, this has not yet been evaluated for the biosynthesis of F_O_.

In this study, microaerobic conditions during the production phase favored F_O_ over riboflavin and resulted in final product titers of 13.91 µmol L^−1^ (5.05 mg L^−1^), which is the highest reported titer for non‐natural F_O_ producers, so far. Finally, a three‐stage purification process allowed for the fractionation of F_O_ from the remaining riboflavin and ultimately yielded a pure product.

## 2 Material and Methods

### 2.1 Microbial Strain Construction and Cultivation Conditions

In this study, an *E. coli* BL21 strain, transformed with the pDB048 plasmid, was used for F_O_ production. The plasmid pDB048 was obtained by insertion of the coding sequence of F_O_‐Synthase (*fbiC*) from *M. rhizoxinica* HKI‐454 [2] into vector pMGE‐T7 [53] at the EcoRV site. Gene expression is controlled by a T7 promotor with a lac operator. A kanamycin resistance cassette (*KanR*) serves as the selection marker.

Strain reactivation from cryo stocks was carried out in 30 mL Luria Broth medium (5 g L^−1^ yeast extract, 10 g L^−1^ peptone, 5 g L^−1^ NaCl). All experiments were conducted in liquid M9 medium (16 g L^−1^ Na_2_HPO_4_ x 12 H_2_O, 0.5 g L^−1^ NaCl, 3 g L^−1^ KH_2_PO_4_, 1 g L^−1^ NH_4_Cl, 10 mL L^−1^ Fe‐citrate solution (6 g L^−1^), 2 mL Zn(CH_3_COO)_2_ x 2 H_2_O solution (4 g L^−1^) and each 0.1 mL L^−1^ of EDTA (84 g L^−1^), CoCl_2_ x 6 H_2_O (25 g L^−1^), MnCl_2_ x 4 H_2_O (150 g L^−1^), CuCl_2_ x 2 H_2_O (15 g L^−1^), H_3_BO_3_ (30 g L^−1^), Na_2_MoO_4_ x 2 H_2_O (25 g L^−1^) solutions). Glycerol as carbon source (10 or 30 g L^−1^ for shake flask cultivation or stirred tank reactors, respectively), MgSO_4_ x 7 H_2_O (0.24 g L^−1^) and kanamycin (30 mg L^−1^) were added after autoclaving.

### 2.2 Pre‐culture

Prior to growth experiments or stirred tank reactor cultivations, a two‐step pre‐cultivation was performed. First, 100 µL of cryo stock was reactivated in 30 mL of LB (10% flask volume) at 37°C and 200 rpm shaking in an orbital shaker with a diameter of 70 mm. The second pre‐culture was inoculated to an OD of 0.01 in M9 media and grown at 37°C overnight. The size of the flask and the shaking frequency depended on the purpose of the following experiment.

### 2.3 Online Respiration Measurement in Shake Flask Cultivation

To enable online off‐gas measurements and calculation of the oxygen transfer rate (OTR) and carbon transfer rate (CTR) during shake flask cultivation, the TOM system from Kühner AG (Birsfelden, Switzerland) was used. To investigate four different levels of oxygen limitation 250 mL non‐baffled Erlenmeyer flasks (Kühner AG, Birsfelden, Switzerland) were filled with 10, 20, 30, and 40 mL of M9 medium in quadruplicate each and incubated at 37°C and 100 rpm (orbital shaking, 50 mm diameter). Every flask was equipped with an online monitoring system for oxygen and carbon dioxide off‐gas measurements. Supplied compressed air contained 0.04% (v/v) CO_2_ and 20.95% (v/v) O_2_. OTR (Equation 1) and CTR (Equation 2) were calculated according to the following equations [54]. Maximum theoretical OTRs were calculated to be 19.9, 11.9, 8.8, and 7.1 mmol L^−1^ h^−1^, respectively.

The inoculum was pre‐grown overnight, and flasks were inoculated to an OD_600_ of 0.1. Cultures were induced with 0.1 mM IPTG once the OTR reached one‐third of the calculated maxima. This online approach avoided interruptions of the cultivations for OD measurements to determine the time point for induction. Samples for HPLC were taken after 24 h of cultivation.

(1) $$\mathrm{OTR}=\frac{{\dot{V}}_{G}}{V_{L}\times V_{norm}}\times \left(y_{O2,in}-\frac{1-y_{O2,in}-y_{CO2,in}}{1-y_{O2,out}-y_{CO2,out}}\times y_{O2,out}\right)$$

(2) $$\mathrm{CTR}=\frac{{\dot{V}}_{G}}{V_{L}\times V_{norm}}\times \left(y_{CO2,out}\times \frac{1-y_{O2,in}-y_{CO2,in}}{1-y_{O2,out}-y_{CO2,out}}-y_{CO2,in}\right)$$

### 2.4 Cultivations in Stirred Tank Reactors

The first scale‐up step after shake flask cultivation was conducted in 7‐L glass reactors (BIOSTAT B‐DCU II, Sartorius Stedim Biotech, Göttingen, Germany) with a working volume of 4 L and controlled via the MFCS‐win software system (Sartorius, Göttingen, Germany). Off‐gas measurement was recorded via BlueSens Blue Vary gas sensors (BlueSens gas sensor GmbH, Herten, Germany). Ammonia (12.5%), sulfuric acid (10%), and antifoam reagent 204 (Merck KGaA, Darmstadt, Germany) were connected externally for pH regulation at 7 ± 0.05 and for foam control during the bioprocess. Two reactors were run in parallel, whereas one had a fixed stirring rate of 469 rpm to limit the oxygen supply and the other had a flexible stirring rate, starting at 300 rpm, that was controlled by the dissolved oxygen tension (DOT) sensor feedback. The DOT regulation was set to 20% to avoid any oxygen limitation during this fermentation run. Glycerol as a carbon source was prepared for a final concentration of 10 g L^−1^ and inoculation was done with a pre‐culture grown overnight in M9 medium. Product formation was induced with 0.1 mM IPTG after 5.5 h of cultivation. The process was stopped once complete glycerol consumption was confirmed.

The final production of F_O_ was performed in a 30‐L stainless steel reactor with in‐situ sterilization (BIOSTAT_D‐DCU Twin MO 20L, Sartorius Stedim Biotech, Göttingen, Germany). The MFCS‐win software system (Sartorius, Göttingen, Germany) was used as a digital control system and for online recording. Off‐gas measurement was recorded with an external Emerson off‐gas‐analysis box (Rosemount NGA 2000, Emerson Process Management GmbH & Co, OHG, Haan, Germany). A tailor‐made fluorescence probe from C‐Technologies (C Technologies, Inc., Bridgewater, USA) was integrated directly into the bioreactor via a Schott Viewport (Schott AG, Mainz, Germany) and connected to an Agilent Cary Eclipse Fluorescence Spectrometer (Agilent Technologies, Santa Clara, USA). Sulfuric acid (12.5%), ammonia (25%), and antifoam reagent 204 (Merck KGaA, Darmstadt, Germany) were connected externally to ensure stable pH control at 7 ± 0.05 and to avoid excessive foaming during the process. As scale‐up criteria, the volumetric power input was chosen accordingly to the following equations (3), (4), (5), (6) [50, 55], and stirring was kept constant at 316 rpm (3‐step Rushton impeller) for limited oxygen supply.

(3) $$\frac{P_{R,7L,aerated}}{V_{R,7L}}=\frac{P_{R,30L,aerated}}{V_{R,30L}}$$

(4) $$u_{g}=\frac{q_{g}}{A_{R}}$$

(5) $$\frac{P_{R,aerated}}{P_{R,unaerated}}={\left(1+375\times \frac{u_{g}}{\sqrt{g\times D_{R}}}\right)}^{-0.5}$$

(6) $$P_{R,unaerated}=Ne\times p\times n_{stirrer}^{3}\times d_{stirrer}^{5}$$

The substrate glycerol was prepared for a final concentration of 30 g L^−1^. Inoculation was done with a pre‐culture grown overnight in M9. Product formation was induced with 0.1 mM IPTG once the DOT decreased to 20%. After 24 h, a second batch of 600 g glycerol was added to the bioreactor. The production process was stopped once DOT and HPLC results confirmed the complete depletion of glycerol.

### 2.5 Downstream Process

The downstream process employed two medium‐pressure liquid chromatography (MPLC) steps using an adsorption resin AmberChrom CG 161 M (DuPont, Wilmington, USA) with different pH, followed by one preparative HPLC step on a C18 reverse phase column with intermediary rotary evaporation and re‐dilution of defined fractions. In detail, the culture broth was first centrifuged, the supernatant filtered with Celpure P1000 (Advanced Minerals, Sheffield, UK) and adjusted to a pH of 3 for the first chromatography step. The acidified supernatant was loaded onto a 1L Amberchrom Column. The process was operated on an ÄKTApilot (Amersham Biosciences, Sweden), controlled by UNICORN software, and elution was monitored at 280, 395, 420 nm wavelengths. Increasing concentrations of methanol (0%, 10%, 50%, and 100%) were used as eluent. The 395 nm peak within the 50% MeOH fraction was collected and concentrated via rotary evaporation (Heidolph Instruments GmbH and Co. KG, Schwabach, Germany). The remaining solid was re‐diluted in bi‐distilled water, and the obtained solution was adjusted to a pH of 8. The intermediary purification proceeded on a 50 mL AmberChrom Column, on an ÄKTAexplorer (Pharmacia Biotech, Uppsala, Sweden), controlled via UNICORN software at 280, 395, and 420 nm. The product was monitored at 395 mm, eluted within the water and the 10% MeOH fraction, and was processed as before. A highly concentrated product solution was injected into a preparative HPLC set‐up (JASCO MD‐2015 plus Multiwavelength Detector and AS‐2050 plus Intelligent Sampler, Jasco International Co, Tokyo, Japan) equipped with a Eurospher II 100–10 C18 column (32 mm ×240 mm, Knauer, Berlin, Germany) and eluted with a gradient of 0.1% formic acid (eluent A) and acetonitrile (eluent B) in 20 min at a flow rate of 20 mL min^−1^. The collected product fraction was concentrated as described before, lyophilized (Alpha 1–4 LDC‐1m, Christ Gefriertrocknungsanlagen GmbH, Osterode, Germany), and stored at 4°C for further purity analysis or until further usage.

### 2.6 NMR Measurement

Lyophilized F_O_ powder was dissolved in DMSO‐d6 (Merck KGaA, Darmstadt, Germany, 10 mg L^−1^), and ^1^H standard spectrum was measured in an Avance III 500 MHz Spectrometer (Bruker Massachusetts, USA) at 500 MHz. Analysis was conducted with TopSpin (Bruker, Massachusetts, USA).

### 2.7 Analytics

Glycerol and metabolites were analyzed via HPLC (X‐LC, Jasco International Co, Tokyo, Japan) with an Aminex HPX‐87H Ion Exclusion main‐column (300 mm × 7.8 mm, 9 µm, Bio‐Rad, Hercules CA, USA). Five millimolar H_2_SO_4,_ with a flow of 0.5 mL min^−1^, was used isocratically as the mobile phase. Peak detection was performed by refractive index and UV (210 nm). Besides fluorescence measurement, F_O_ formation was additionally quantified offline via UHPLC (Vanquish, Thermo Fisher Scientific, Waltham MA, USA), equipped with a Luna Omega PS C18 column (150 mm × 2.1 mm, 3 µm, Phenomenex, Torrance CA, USA). The mobile phase consisted of eluent A 0.1% formic acid in water and eluent B 0.1% formic acid in acetonitrile, with a flow of 0.15 mL min^−1^ and mixed in a gradient from 15% B to 100% B in 15 min and then back to 15% B in 16 min. Peak detection was monitored via fluorescence detector (ex 390 nm, em 470 nm) and DAD (395 nm).

## 3 Results and Discussion

A main challenge for efficient F_O_ production is the competing formation of riboflavin through the interconnection of the biosynthetic pathways. We approached this challenge on both the upstream and downstream levels.

### 3.1 Oxygen Limitation Shifts Production From Riboflavin to F_O_

**FIGURE 2:**
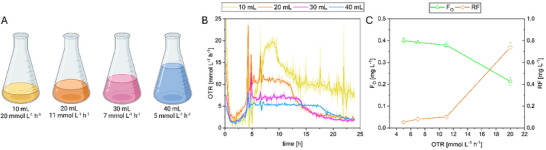
Evaluation of different oxygen availabilities on F_O_ and riboflavin biosynthesis by *E. coli* pDB048. Cells were grown in different volumes of M9 medium with glycerol as carbon source in shake flasks as quadruplicates. Uniform cultivation conditions were: 37°C, 100 rpm, 50 mm shaking diameter, 250 mL flasks, in a shaker equipped with the TOM off‐gas monitoring setup. Off‐gas O_2_ and CO_2_ values were measured and the OTR was calculated (B). The measured OTR_max,E_ and the corresponding filling volume are shown in (A). Titers of riboflavin (RF) and F_O_ were measured via UHPLC after 24 h at the end of the cultivation (C).

To investigate the effect of different levels of oxygen supply on F_O_‐formation by *E. coli* pDB048, four standard 250 mL Erlenmeyer flasks for the TOM respiration monitoring device were filled with 10, 20, 30, and 40 mL of culture media and used for parallel cultivation (Figure 2A). Thereby, the different filling volumes allowed to define theoretical maximum OTRs (OTR_max_) for the different cultivations (20, 12, 9, and 7 mmol L^−1^ h^−1^, respectively). Off‐gas composition was measured online and the actual OTR (OTR_E_) and CTR were calculated (Figure 2B). F_O_ biosynthesis was induced when approximately two‐thirds of the theoretical OTR_max_ was reached in each culture setup, respectively. After 24 h of cultivation, titers for riboflavin and F_O_ were determined (Figure 2C). As previously noticed for other strains, a shift in redox cofactor formation was observed. Riboflavin formation drastically decreased from approximately 0.7 ± 0.04 mg L^−1^ (OTR_max,E_ 20 mmol L^−1^ h^−1^) to 0.05 ± 0.01 mg L^−1^ (OTR_max,E_ 5 mmol L^−1^ h^−1^) from cultures without oxygen limitation to conditions with lower OTR. On the other hand, F_O_ production increased (Figure 2C). No distinct difference in the F_O_ titer was observed between the 20, 30, and 40 mL cultures. These results might be due to the intrinsic oxygen sensitivity of the F_O_ synthase [42] and thus microaerobic conditions might promote deazaflavin formation. However, the product titer for F_O_ was still below 0.5 mg L^−1^ (0.4 ± 0.01 mg L^−1^, 1.1 ± 0.03 µmol L^−1^). Thus, the fraction of carbon source (glycerol) directed into F_O_ biosynthesis was low under these conditions. For the active production phase of the culture (from induction to exhaustion of the substrate as indicated from the OTR signal), productivities of approximately 178, 136, 121, and 82 nmol L^−1^ h^−1^ (from high to low oxygen availability) were reached in the different cultures.

A recent study investigated the effect of different *E. coli* host strains, F_O_ synthase enzymes (FbiC from *M. smegmatis* and *Streptomyces coelicolor* and CofG/H from *Methanocaldococcus jannaschii* and *Nostoc punctiforme*) and media composition on F_O_P (the phosphorylated form of F_O_) formation in shake flask [36]. The authors demonstrated that high F_O_ synthesis was crucial for further phosphorylation efficiency in F_O_P and concluded that increased *fbic* expression resulted in higher F_O_P production. Interestingly, the exchange of glucose with glycerol in M9 medium enhanced the F_O_P titer. However, cultivation on terrific broth yielded F_O_P titers that were more than 50 times higher (1.24 µmol L^−1^ in TB and 4.8 nmol L^−1^ in M9). Another study from 2021 reported that F_420_ biosynthesis in *E. coli* was heavily influenced by the choice of carbon source [24]. Positive influences on F_O_P and F_420_ production cannot be directly translated to F_O_ production, since in those studies excessive F_O_ production is usually avoided to prevent intermediate loss into the supernatant [23]. However, F_O_ production and production of its derivatives, F_420_ and F_O_P, may still be affected by similar mechanisms. In our study, the highest titer of F_O_ in shake flask with glycerol as a carbon source (1100 nmol L^−1^, average productivity 136 nmol L^−1^ h^−1^) was considerably higher than the titer published by Graham and colleagues (160 nmol L^−1^, in M9 with glucose). On the other hand, the highest average productivity for our work was found in a culture with no oxygen limitation (OTR_max,E_ of 20 mmol L^−1 ^h^−1^, productivity 178 mmol L^−1 ^h^−1^). However, since F_O_ formation was not followed online, the overall productivities do not allow to judge on effective maximum productivities for the different oxygenation levels. We decided against the use of a complex medium, as we desired defined conditions within our process, especially in foresight of a targeted media optimization for higher cell densities in the future. Furthermore, certain compounds hinder or prolong the downstream processing of the fermentation broth. Our goal always included an efficient scale‐up and downstream design, and therefore the complexity of the whole process was to be kept concise. The target adjustment here was focused on the oxygen limitation, which was not investigated before and has been proven to have a great effect on the productivity within the chosen media conditions. However, further investigation into media optimization would be a worthwhile extension of our studies.

For example, as tyrosine is one of the two precursor molecules during F_O_ formation, enhanced supply could lead to higher flux directed into the condensation reaction. But different studies presented contradictory results regarding this. In one study, a positive effect on final F_O_ titers was confirmed for tyrosine supplementation (from 160 nmol L^−1^ to 640 nmol L^−1^, [39]). Though, in another study, the same approach did not result in a similar outcome [36].

In 2002, Isabelle and colleagues published a comparative study evaluating F_O_ and F_420_ titers within cells and in cell supernatant in shake flask produced by various wildtype microbial strains [20]. Two different media that were rich in nutrients were used, depending on the host strain's preferences. The highest levels of F_O_ in culture supernatants were 2.6 µmol L^−1^ (*Streptomyces flocculus*), 1.24 µmol L^−1^ (*M. smegmatis*) and 1.75 µmol L^−1^ (*Rhodococcus opacus* Rb1). Noteworthy, these strains may not be pathogenic, but in comparison to *E. coli*, current tools for metabolic engineering are more time‐consuming or limited, cell growth and maximum cell densities are lower, and scale‐up may be difficult.

### 3.2 Scale‐Up Into a 30‐L Stirred Tank Reactor

**FIGURE 3:**
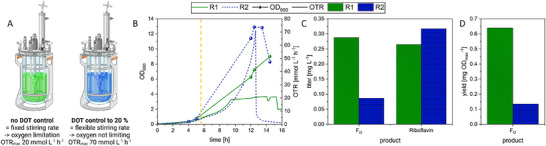
F_O_ Fermentation with *E. coli* pDB048 under oxygen limited (R1, green) or unlimited (R2, blue) conditions in 7‐L glass bioreactors. Non‐limiting conditions were implemented through a DOT regulation at 20%, whereas oxygen limited conditions were reached through a fixed stirring rate (A). OD_600_ was measured offline and OTR and was calculated from online off‐gas measurements (A). F_O_ and riboflavin titers were measured via UHPLC and shown are the final titers of the bioprocess (B). The F_O_ yield was calculated for the total amount of product and the OD_max_ for both reactors respectively (D). Induction of F_O_ formation through IPTG addition is marked with the dashed orange line.

After evaluating the influence of the oxygen supply on the riboflavin and F_O_ synthesis, a scale‐up strategy was designed based on the OTR as scale‐up criterion. Microaerophilic conditions are especially convenient for upscaling processes, as oxygen supply is usually one of the key limiting factors. A first scale‐up was conducted to two parallel 7‐L stirred tank reactors with a working volume of 4 L to confirm a positive effect of oxygen limitation through the application of a fixed stirring rate, which limits the OTR_max_ to approximately 20 mmol L^−1^ h^−1^ in comparison to an unlimited dissolved oxygen regulation to DOT = 20% through a variable stirring rate (OTR_max_ ∼ 70 mmol L^−1^ h^−1^) (Figure 3A,B). This strategy was chosen with the motivation to reach higher overall cell densities in combination with the induction of F_O_ formation only a few minutes before reaching oxygen limitation.

These preliminary bioprocesses confirmed slightly enhanced F_O_ titers and a strong reduction of riboflavin production without DOT regulation (Figures 3C and S1). Noteworthy, the oxygen limitation also resulted in a 4‐fold higher biomass related yield of F_O_ (Figure 3D). In a subsequent 7‐L fermentation under equivalent oxygen limitation, we prolonged the F_O_ production over the course of 24 h with higher initial glycerol concentrations leading to a 6‐fold increase in final F_O_ titers compared to the initial 7‐L bioreactor (from 0.3 to 1.8 mg L^−1^, Figure S2).

**FIGURE 4:**
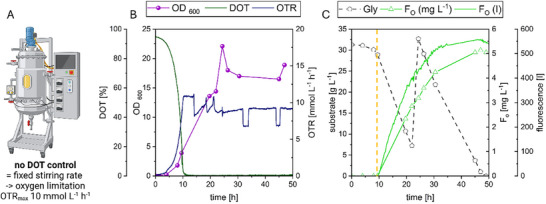
F_O_ production with *E. coli* pDB048 under oxygen‐limited conditions and with glycerol as carbon source in a stainless‐steel reactor (A). Dissolved oxygen tension within the fermentation broth was measured (DOT, green). Oxygen Transfer Rate (OTR, blue) was calculated from external online off‐gas measurements. OD_600_ (purple) was measured offline (B). Glycerol depletion (Gly, black), and product formation (F_O_, green triangles) were measured offline. For additional metabolite concentrations see Figure S3. Additionally, online F_O_ monitoring was carried out via fluorescence measurements at 400 nm excitation and 466 nm emission (green line) (C). Induction of F_O_ formation through IPTG addition is marked with the dotted orange line. An independent repetition is given in Figure S4.

Finally, the F_O_ biosynthesis was scaled up to a 30‐L stainless steel bioreactor with a working volume of 20 L. Oxygen‐limited conditions were implemented by setting a fixed stirring rate of 316 rpm and a constant airflow of 5 L min^−1^ to reach an OTR_max,E_ of 10 mmol L^−1^ h^−1^ (Figure 4, independent repetition is shown in Figure S4). The higher oxygen limitation was chosen to further reduce riboflavin titers, while F_O_ titers remained fairly stable during the experiments with limited and unlimited DOT conditions in the 7‐L bioreactor. During the previous flask experiment, the culture with an OTR_max_ of 10 mmol L^−1^ h^−1^ showed the highest productivity. This time, F_O_ synthesis was induced 9.6 h after inoculation when approximately two‐thirds of the maximum OTR for these conditions was reached, and production was followed online by an inserted fluorescence probe (Figure 4A). Shortly after induction, oxygen limitation was reached, indicated by a DOT of zero. The initial glycerol was depleted after 24 h, and fresh glycerol was added to extend the fermentation process to 48 h (Figure 4B). The maximum glycerol consumption rates for both batches were approximately 1.8 g L^−1^ h^−1^. However, during the second feeding period, the measured online fluorescence (Figure 4C) indicated that no further product was biosynthesized after approximately 33 h. The remaining carbon source was utilized for neither F_O_ nor biomass production, indicating the presence of a second substrate limitation for growth. However, as remaining glycerol was conflicting with the following downstream purification process, it was of utmost importance that all glycerol be consumed prior to the continuation of the process.

Interestingly, of the four analyzed organic compounds acetic acid, succinic acid, lactic acid, and ethanol, only the lactic acid production changed after 33 h (Figure S3). At oxygen limitation, the CTR and the OTR were stable (Figure 4B), only to be interrupted by the effect of anti‐foam dosing into the reactor. Both rates reached maximum values (7 and 11 mmol L^−1^ h^−1^, respectively) when the oxygen limitation was reached, decreased shortly after, and remained at the lower level (5.5 and 9 mmol L^−1^ h^−1^, respectively) throughout the remaining process.

The final F_O_ titer within this process was 13.91 µmol L^−1^ (5.05 mg L^−1^) in approximately 20 L of fermentation broth. The side product riboflavin only reached concentrations of 1.86 µmol L^−1^. With regards to the online fluorescence data, it can be concluded that F_O_ synthesis correlates with cell growth, and this is equivalent to an average productivity of 548 nmol L^−1^ h^−1^ if a timeframe from 9.6 h (induction) up to 35 h (cease of production and end of active phase) is considered. Isabelle and colleagues proceeded their investigation of wildtype F_O_ and F_420_ producers and transferred selected microbial strains into bioreactors as well [20]. Here, the highest F_O_ titers were found in the supernatant of the *M. smegmatis* fermentation processes (6 µmol L^−1^). The whole process lasted 70 to 90 h, but only low productivity F_O_ was found for the first 50 h. While the active phase of the bioprocess had a productivity of around 300 nmol L^−1^ h^−1^ (if we consider an F_O_ biosynthesis for 20 h), the extensive production lag phase of 50 h would prevent a biotechnological exploitation. In another work, Kern et al. described the early appearance of a greenish compound during the *Methanobacterium thermoautotrophicum* fermentation process, which was later confirmed to be F_O_ [25]. Approximately 25 mg F_O_ was harvested from the 12 L fermentation (5.73 µmol L^−1^), however not many process parameters are reported.

For better comparison, all previously discussed data from both shake flask and bioreactor experiments are summarized in Table 1. This comparison highlights that our optimized bioprocess design with an engineered *E. coli* for heterologous F_O_ production can compete with previous work and considerably surpasses the studies utilizing natural producers in a bioreactor setup. Noteworthy, we achieved our results without extensive media optimization, but with a focus on oxygen availability as a critical factor influencing the bioproduction. However, since our online fluorescence monitoring suggests a direct correlation of F_O_ synthesis with growth, an extension of the growth phase could potentially lead to an extension of the F_O_ production phase. Certainly, the fine‐tuning of effects of oxygen on the FbiC enzyme and the redox‐active product needs to be investigated in more detail.

**TABLE 1:** Comparative summary of different studies on F_O_ synthesis.

Strain	Variant	Medium	Product titer (mg L^−1^)	Product titer (nmol L^−1^)	Productivity (nmol L^−1^ h^−1^)	Study
**Shake flask**						
*E. coli*	FbiC	M9 (glycerol)	0.40	1100 ± 30	136	This study
*E. coli*	FbiC	M9 (glucose)	0.06	160	80	Graham et al. [39]
		M9 (glucose) + tyrosine	0.23	640	320	Graham et al. [39]
		LB	0.11	290	145	Graham et al. [39]
*S. flocculus*	Wt	MRS^1^	0.94	2580	n.d.	Isabelle et al. [20]
*M. smegmatis*	Wt	MRS^1^	0.45	1240	n.d.	Isabelle et al. [20]
*R. opacus* Rb1	Wt	MRS^1^	0.64	1750	n.d.	Isabelle et al. [20]
**Bioreactor**						
*E. coli*	FbiC	M9 (glycerol)	5.05	13,900	548	This study
*M. thermo‐autotrophicum*	Wt	A1^2^	2.08	5740	n.d.	Kern et al. [25]
*M. smegmatis*	Wt	MRS^1^	2.18	6000	150–300	Isabelle et al. [20]

*Note*: The productivity was calculated from given process parameters if not stated directly in the results. Not defined (n.d.) parameters were not accessible through the given information within the published study.

^1^ MRS medium: 40 g L^−1^ glucose, 15 g L^−1^ yeast extract, 15 g L^−1^ soy peptone, 1.75 g L^−1^ NaH_2_PO_4_ x 4 H_2_O, 0.04 g L^−1^ ferric ammonium citrate, adjusted to pH 7.

^2^ A1 medium: A1 medium published by Andrews and Presnell [56]: 5 g L^−1^ lactose, 20 g L^−1^ tryptone, 5 g L^−1^ NaCl_2_, 0.5 g L^−1^ salicin, 1 mL L^−1^ Triton X‐100 but supplemented with 2.4 mg L^−1^ NiCl_2_ x 6 H_2_O.

### 3.3 Downstream Processing: Obtaining Pure F_O_

**FIGURE 5:**
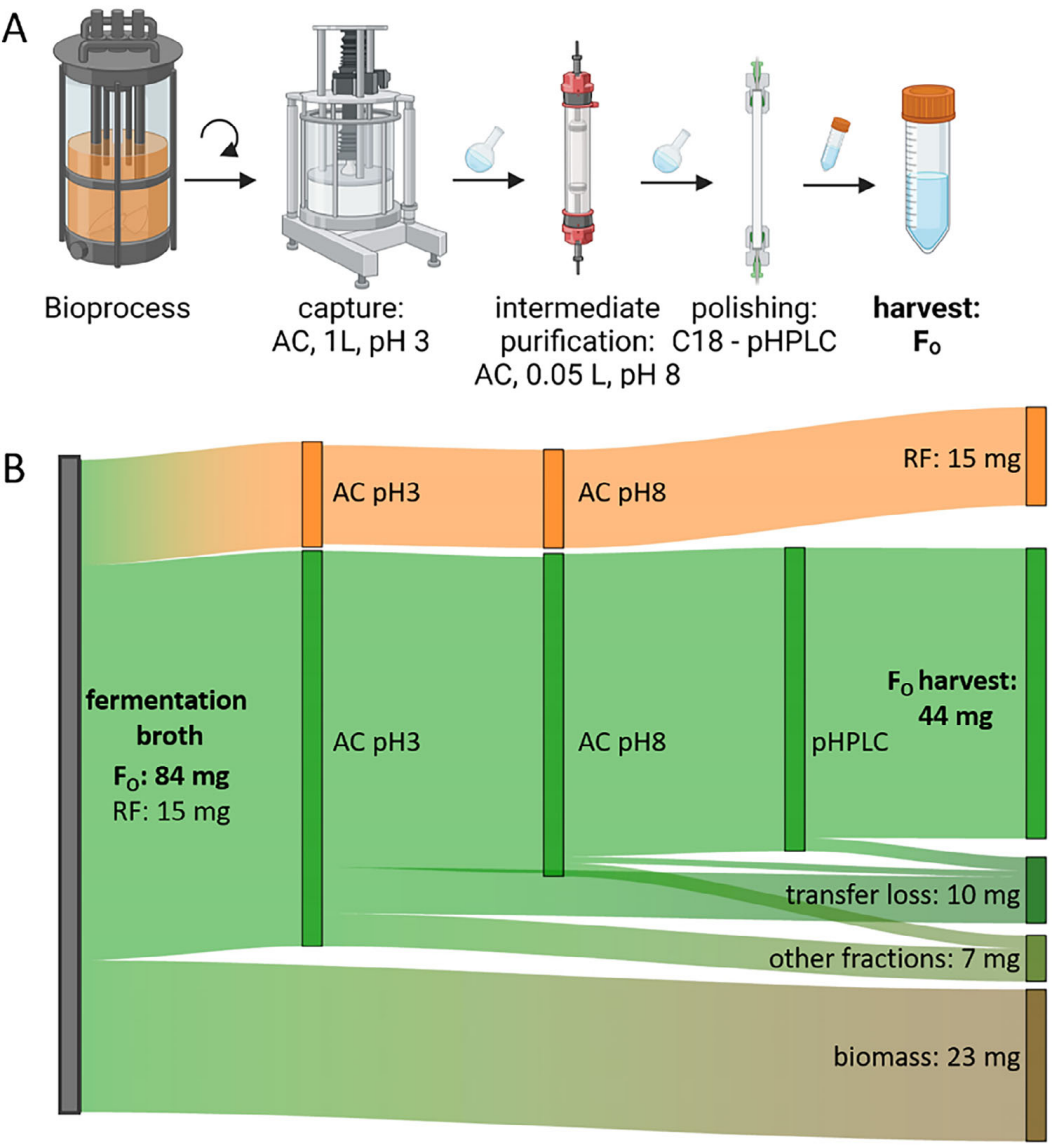
Overview of the different downstream processing steps required for F_O_ product purification from crude fermentation broth to the collection of a pure product. Schematic figures represent the station of the downstream process adsorption chromatography (AC) and preparative HPLC (pHPLC), intermitted by centrifugation (arrow), rotary evaporation (small round flask), and lyophilization (small falcon tube, A). The Sankey diagram below represents the fractions of riboflavin (RF, orange) and F_O_ (green). The amount of RF and F_O_, labeled in the graph are representative for one bioprocess and the corresponding downstream process. Riboflavin is separated from F_O_ after the intermediate purification at pH 8.

Concomitantly with optimizing the F_O_ bioprocess, we designed an efficient downstream purification protocol to obtain the pure compound for further usage. The final downstream process for F_O_ purification consists of three chromatography steps, intermitted by rotary evaporation for fraction concentration and preparation for the corresponding next chromatography step (Figure 5). During this process, separation of F_O_ from structurally very similar metabolites like riboflavin was prioritized.

As described in 1983 by Kern et al., F_O_ is membrane permeable and can be harvested from the crude cell broth without cell lysis [25]. This is beneficial for online product monitoring and simplifies the first step of the downstream process. The biomass can be discarded after separation, which was achieved via centrifugation. Centrifuged and filtered fermentation supernatant was adjusted to pH 3 and loaded onto a 1‐L chromatography column filled with AmberChrom CG 161 M resin. Elution was conducted with increasing concentrations of MeOH in bi‐distilled water, and F_O_ was captured within the second peak fraction in 50% MeOH (Figure S5). Riboflavin, the main side product of F_O_ biosynthesis, shares an overall similar molecular structure but distinct differences: N‐5 is replaced against a carbon atom, C‐7 and C‐8 are demethylated, and C‐7 is hydroxylated in F_O_ [57, 58, 59]. At pH 3, the polarity of both compounds is similar, and therefore, both eluted in the second 50% MeOH fraction (Figure S5). However, other fermentation products and metabolites were successfully separated. To separate riboflavin and F_O_, another chromatography step was introduced subsequently. After the removal of MeOH residues in a rotational evaporator, the collected residue was suspended in bi‐distilled water and the pH was set to 8 prior to a second adsorption chromatography step. At pH 8, the structural differences yield different physiochemical properties for riboflavin and F_O_. The 8‐hydroxy group of F_O_ deprotonates, leading to the formation of a delocalized phenolate above pH 6, induces increased chromatographic discrimination of both analytes on the apolar column material (Figure S6), whereby the majority of F_O_ elutes before riboflavin. Eventually, the collected material was purified via preparative HPLC on a C18‐column. After lyophilization, the purity of the product was confirmed via ^1^H NMR measurement. The 500 MHz ^1^H NMR spectrum (3.46; 3.6; 3.66/3.64; 4.24; 4.49; 4.65; 4.67; 4.79; 4.96/4.95; 5.13/5.12; 7.04/7.02; 7.39; 8.01/8.03; 8.88; 10.97; 11.25 ppm, Figure S7) was in accordance with the results published previously [29].

A schematic view of the full downstream process is shown in Figure 5. In the presented example data, the concentration of F_O_ within the fermentation broth was determined to be approximately 4 mg L^−1^ F_O_. About 53% of the product was harvested as pure F_O_. Product loss during purification can be attributed to different reasons: 27% of loss is attributed to the biomass separation, during which no washing was included, and potential degradation during preparation for the chromatography procedure. More product loss is attributed to the lengthy purification process and F_O_ elution within non‐collected fractions (Figures S5 and S6).

A previously published F_O_ purification comprised a total of four chromatography steps, including an adsorption chromatography via an Amberlite XAD‐4 column, subsequent anion exchange chromatography via a sephadex QAE A‐25 column, another adsorption chromatography via the previously used column setup and a final step via preparative HPLC on a Lichrosorb RP 18/10 µm column [25]. Our process requires one step less during intermediate purification, thus potentially minimizing the loss of product during the workup. Moreover, our process enabled the confirmed separation of contaminating compounds like riboflavin from the target compound.

## 4 Conclusion

In this study, we established a robust bioprocess for F_O_ production with *E. coli* and performed a successful scale‐up to pilot scale to obtain preparative amounts of F_O_. We evolved the bioprocess from shake flask, over 7‐L glass to 30‐L stainless steel pilot bioreactors with controlled oxygen and pH conditions. Enhanced starting concentration of glycerol as a carbon source led to extended F_O_ synthesis, supported by pH control at 7 ± 0.05. Most pronouncedly, the effect of oxygen limitation during the heterologous production of FbiC and biosynthesis of F_O_ not only proved beneficial for F_O_ synthesis but at the same time suppressed riboflavin synthesis. Furthermore, online fluorescence monitoring of product titers within the bioreactor showed that F_O_ biosynthesis is linked to cell growth. The fermentation process presented here yielded a F_O_ titer of 13.91 µmol L^−1^ (or 5.05 mg L^−1^), which, to our knowledge, is the highest reported thus far. It should be highlighted that simultaneously to the bioprocess optimization, it was very important to develop a robust purification process that delivered pure F_O_. In this regard, a three‐step chromatography process utilizing the effects of different pH and protonation states of the compounds was necessary to discriminate and separate F_O_ from other flavin congeners like riboflavin. NMR analysis confirmed the high purity of the harvested F_O_, which is now available for further studies.

The general research focus on and applications for F_O_ are still rather low in comparison to its phosphorylated form F_O_P and the full cofactor F_420_, with much more active research regarding their functionality and activity in biotechnologically relevant enzymatic reactions. That said, a vast range of F_420_‐dependent enzymes has not been characterized yet. To simplify the assessment of these enzymes—since the F_420_ cofactor is fairly hard to obtain—it would be convenient to enable investigations with a cofactor like F_O_ that is now available in higher abundance and can be reproduced conveniently, without extensive strain handling. Furthermore, there is a great possibility that our findings for improved F_O_ biosynthesis in *E. coli* can be advantageous for the future full biosynthesis process of F_420_ in *E. coli* itself, since F_O_ as an F_420_ precursor is needed in any F_420_ biosynthesis process. Fraaije et al. already showed that the F_O_ biosynthetic step can be crucial for further deazaflavin synthesis and that some species like *Saccharomyces* are unable to heterologously produce F_O_, even after extensive engineering, and external supply of the precursor is necessary for any further investigation [36, 37]. Last but not least, F_O_ by itself is increasingly coming into the focus of research. New roles in direct biocatalysis or different functions were proposed. It was recently found to be an important factor in inter‐kingdom communication, and it can be speculated, that this finding opens up a whole new area of investigations, not as a pure redox cofactor but as a signaling molecule [38]. In conclusion, an increased availability of F_O_ can be of great importance for further research, and further optimization of the bioproduction process is a worthwhile investment on many sides.

## Author Contributions

**Annika Lenić**: conceptualization, methodology, investigation, formal analysis, data curation, writing – original draft preparation. **Bettina Bardl**: investigation, reviewing and editing. **Florian Kloss**: investigation, formal analysis, reviewing, and editing. **Gundela Peschel**: methodology, reviewing and editing. **Ivan Schlembach**: methodology, data curation, reviewing, and editing. **Gerald Lackner**: methodology, resources, reviewing and editing. **Lars Regestein**: conceptualization, methodology, data curation, reviewing, and editing. **Miriam A. Rosenbaum**: supervision, conceptualization, visualization, funding acquisition, reviewing, and editing.

## Conflicts of Interest

The authors declare no conflicts of interest.

## Supporting information



Supporting information

## Data Availability

The data that support the findings of this study are available within this article and its supplementary materials or are available upon requesting it from the corresponding authors.
