# Supplementary material for: Pilot Scale Production of a F420 Precursor Under Microaerobic Conditions
Source: Biotechnol J. 2025 Mar 18;20(3):e70002. doi: 10.1002/biot.70002 (PMC11917521; doi:10.1002/biot.70002)
Supplement: Supplementary file 1 — Supporting information [file BIOT-20-e70002-s001.docx]

**Supplementary Information (SI):**

**Pilot scale production of a F_420_ precursor under microaerobic conditions**

Annika Lenić^1,2^, Bettina Bardl^1^, Florian Kloss^3^, Gundela Peschel^1^, Ivan Schlembach^1^, Gerald Lackner^4^, Lars Regestein^1,2^ and Miriam A. Rosenbaum^1,2^

^1^Bio Pilot Plant, Leibniz-Institute for Natural Product Research and Infection Biology, Hans-Knöll-Institute, Jena, Germany

^2^ Faculty of Biological Sciences, Friedrich-Schiller-University, Jena, Germany

^3^Transfer Group Anti-infectives, Leibniz-Institute for Natural Product Research and Infection Biology, Hans-Knöll-Institute, Jena

^4^Chair of Biochemistry of Microorganisms, Faculty of Life Science: Food, Nutrition and Health, University Bayreuth, Bayreuth, Germany

[annika.lenic@leibniz-hki.de](mailto:annika.lenic@leibniz-hki.de)

[bettina.bardl@leibniz-hki.de](mailto:bettina.bardl@leibniz-hki.de)

[florian.kloss@leibniz-hki.de](mailto:florian.kloss@leibniz-hki.de)

[gundela.peschel@leibniz-hki.de](mailto:gundela.peschel@leibniz-hki.de)

[ivan.schlembach@leibniz-hki.de](mailto:ivan.schlembach@leibniz-hki.de)

[gerald.lackner@uni-bayreuth.de](mailto:gerald.lackner@uni-bayreuth.de)

[lars.regestein@leibniz-hki.de](mailto:lars.regestein@leibniz-hki.de)

[miriam.rosenbaum@leibniz-hki.de](mailto:miriam.rosenbaum@leibniz-hki.de)

Author: Miriam A. Rosenbaum, Beutenbergstr. 11a, 07745 Jena, Phone: +49-3641-5321120, Email: [miriam.rosenbaum@leibniz-hki.de](mailto:miriam.rosenbaum@leibniz-hki.de)

Running title: Microaerobic bioproduction of a F_420_ precursor

This Supplementary Information contains:

[Supplementary Figure 1: Substrate consumption and metabolite formation of E. coli pDB048 during F_O_ synthesis in 7-L glass bioreactors with a working volume of 4 L. 3](#_Toc191367476)

[Supplementary Figure 2: F_O_ bioprocess with E. coli pDB048 in a 7-L glass bioreactor with a working volume of 4 L and an enhanced initiate glycerol concentration. 3](#_Toc191367477)

[Supplementary Figure 3: Organic metabolites (A) and flavin products (B) of E. coli pDB048 during F_O_ synthesis in 30-L stainless steel pilot bioreactors with a working volume of 20 L. 4](#_Toc191367478)

[Supplementary Figure 4: Repetition of F_O_ biosynthesis process with E. coli pDB048 in a 30-L stainless steel pilot bioreactor with a working volume of 20 L. 4](#_Toc191367479)

[Supplementary Figure 5: Exemplary chromatogram of an AmberChrom chromatography at pH 3. 5](#_Toc191367480)

[Supplementary Figure 6: Exemplary chromatogram of an AmberChrom chromatography at pH 8. 5](#_Toc191367481)

[Supplementary Figure 7: ^1^H NMR spectrum of purified F_O_, dissolved in DMSO-d^6^ (3.33 and 2.51/2.50 ppm). 6](#_Toc191367482)


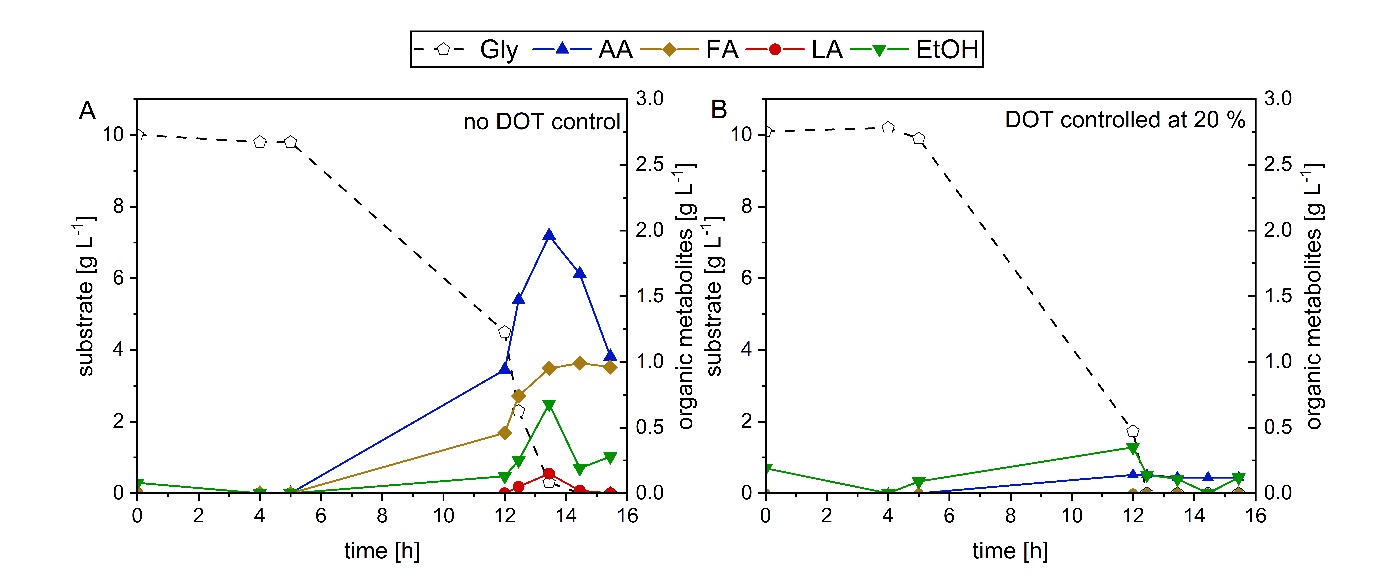


**Supplementary Figure 1:** Substrate consumption and metabolite formation of E. coli pDB048 during F_O_ synthesis in 7-L glass bioreactors with a working volume of 4 L. Bioprocesses were performed with limited (A) or unlimited (B) oxygen availability. All organic metabolites (acetic acid, AA blue; formic acid, FA, brown; lactic acid, LA, red and ethanol, EtOH, green) and the substrate glycerol (Gly, black) were analyzed via HPLC measurements.


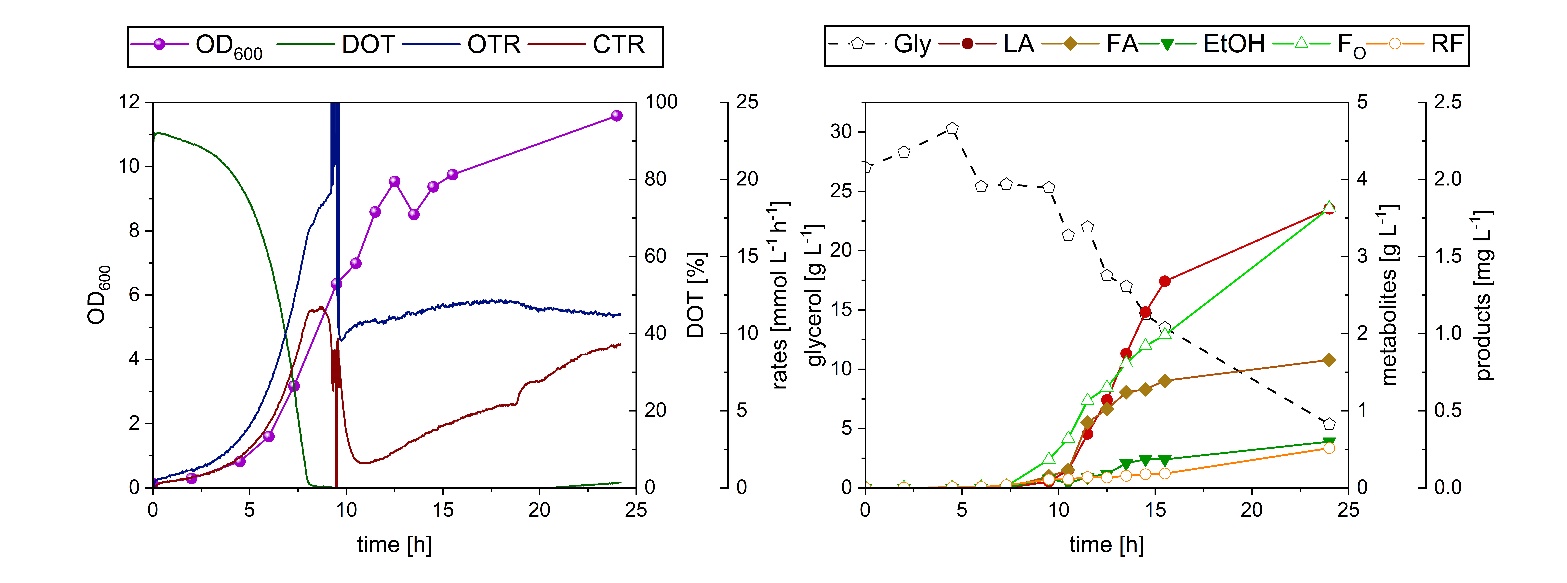


**Supplementary Figure 2:** F_O_ bioprocess with E. coli pDB048 in a 7-L glass bioreactor with a working volume of 4 L and an enhanced initiate glycerol concentration. The process was performed under oxygen limited conditions. DOT was measured online and OTR and CTR were calculated from external off gas monitoring (A). Glycerol (Gly, black) and all organic metabolites (lactic acid, LA, red; formic acid, FA, brown; ethanol, EtOH, green) were analyzed via HPLC measurement (B). The flavin products F_O_ (neon green) and riboflavin (RF, orange) were analyzed via UHPLC (B). Product formation was induced with IPTG after approx. 7.3 h of cultivation.


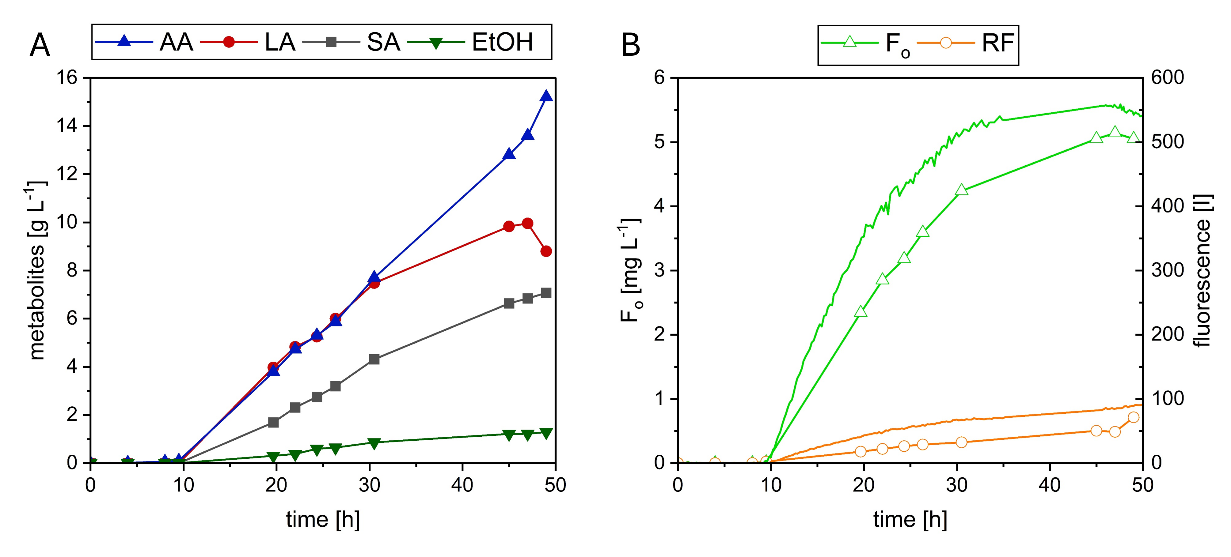


**Supplementary Figure 3:** Organic metabolites (A) and flavin products (B) of E. coli pDB048 during F_O_ synthesis in 30-L stainless steel pilot bioreactors with a working volume of 20 L. All organic metabolites (acetic acid, AA blue; lactic acid, LA, red; succinic acid, SA, grey and ethanol, EtOH, green) in (A) were analyzed via HPLC measurement. F_O_ and riboflavin (RF) in (B) were analyzed via UHPLC (offline, lines with symbols) and online fluorescence measurements (lines without symbols) at different wavelengths (F_O_: Excitation 400 nm, Emission 466 nm; riboflavin: Excitation 450 nm, Emission 525 nm) directly within the tank bioreactor


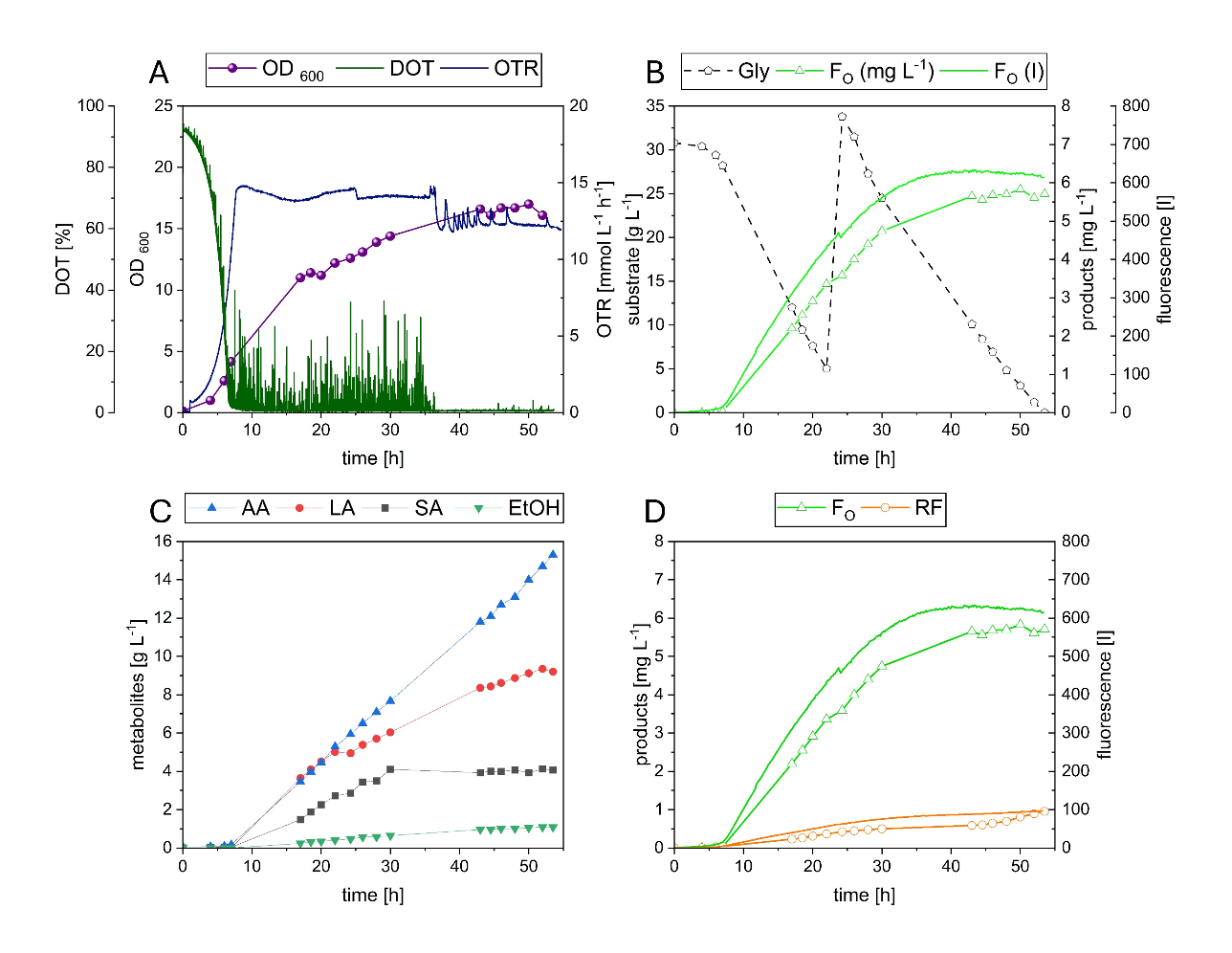


**Supplementary Figure 4**: **Repetition of** F_O_ biosynthesis process with E. coli pDB048 in a 30-L stainless steel pilot bioreactor with a working volume of 20 L. Growth and activity (A), substrate consumption and flavin product formation (B and D) and organic metabolite production (C) were monitored during the process. Product formation was induced after 6 h. (A) Growth was monitored via OD_600_ (purple dots), dissolved oxygen tension (DOT, green line) and oxygen transfer rate (OTR, blue line). (B) Glycerol consumption (Gly, black pentagon) and F_O_ production (light green triangle and light green line) and (C) organic metabolites (acetic acid, AA blue triangle; lactic acid, LA, red dot; succinic acid, SA, grey square and ethanol, EtOH, green upside-down triangle) were analyzed via HPLC measurement. (D) The production of F_O_ (light green) and riboflavin (RF, orange) were additionally measured online, directly within the tank bioreactor with a fluorescence probe (light green and orange lines; F_O_: Excitation 400 nm, Emission 466 nm; riboflavin: Excitation 450 nm, Emission 525 nm) and are shown in comparison to the corresponding offline measurements (F_O_, light green triangle and RF, orange diamond).


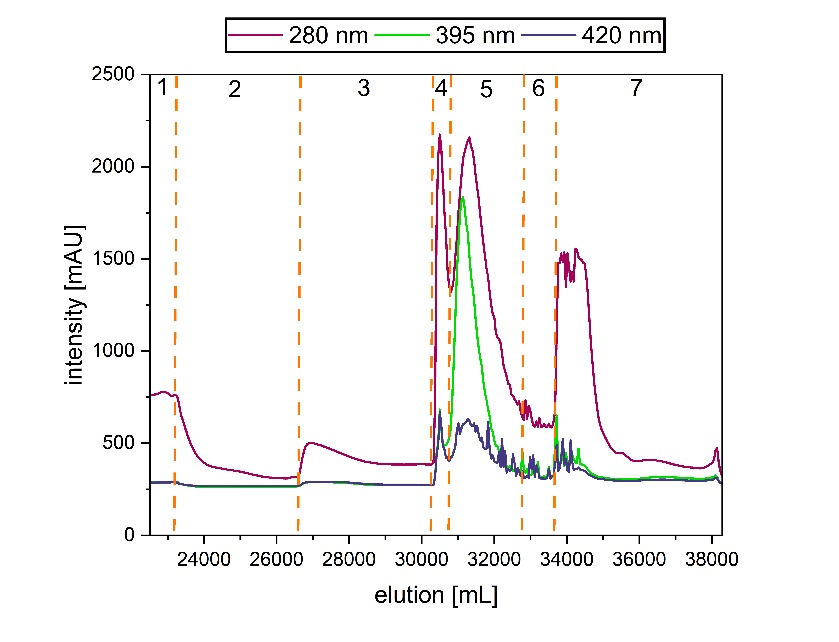


**Supplementary Figure 5:** Exemplary chromatogram of an AmberChrom chromatography at pH 3. Fractions are divided in 1: last residue of loading, 2: washing fraction with water, 3: washing fraction with 10 % MeOH, 4, 5, 6: different fractions of 50 % MeOH and 7: washing fraction with 100 % MeOH. Three different wavelengths 280 (general protein), 395 (F_O_) and 420 (riboflavin) nm were monitored to separate protein (280 nm) fractions and to collect different peak fractions eluting within one concentration of solvent. F_O_ and riboflavin both elute during the second 50 % MeOH fraction (5) at this pH.


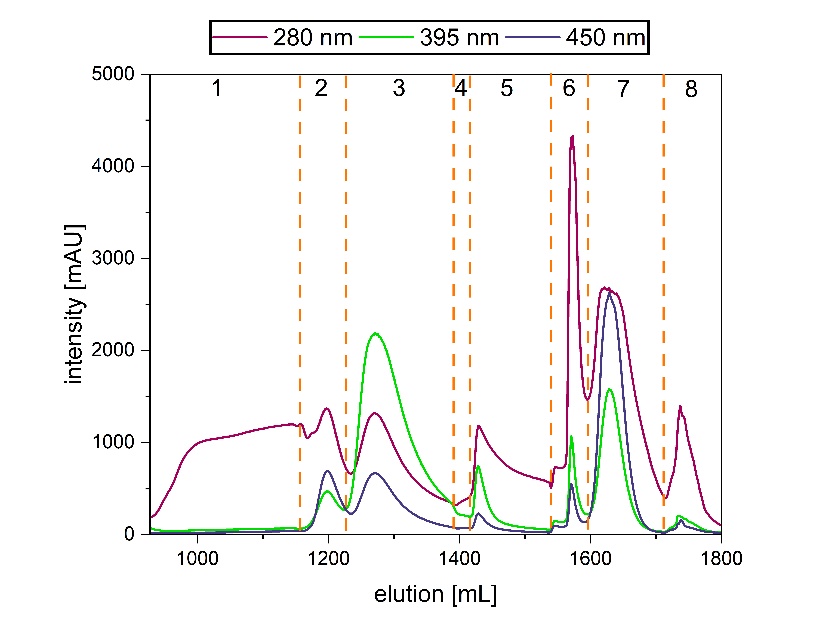


**Supplementary Figure 6:** Exemplary chromatogram of an AmberChrom chromatography at pH 8. Fractions are divided in 1: loading, 2, 3, 4: fractions with water, 5: fraction with 10 % MeOH, 6, 7: different fractions of 50 % MeOH and 8: washing fraction with 100 % MeOH. Three different wavelengths 280 (general protein), 395 (F_O_) and 420 (riboflavin) nm were monitored to separate fractions and collect different peak fractions eluting within one concentration of solvent. At this pH, F_O_ elutes during the second water fraction (3) and the 10 % MeOH fraction (5), while riboflavin elutes during the second 50 % MeOH fraction.


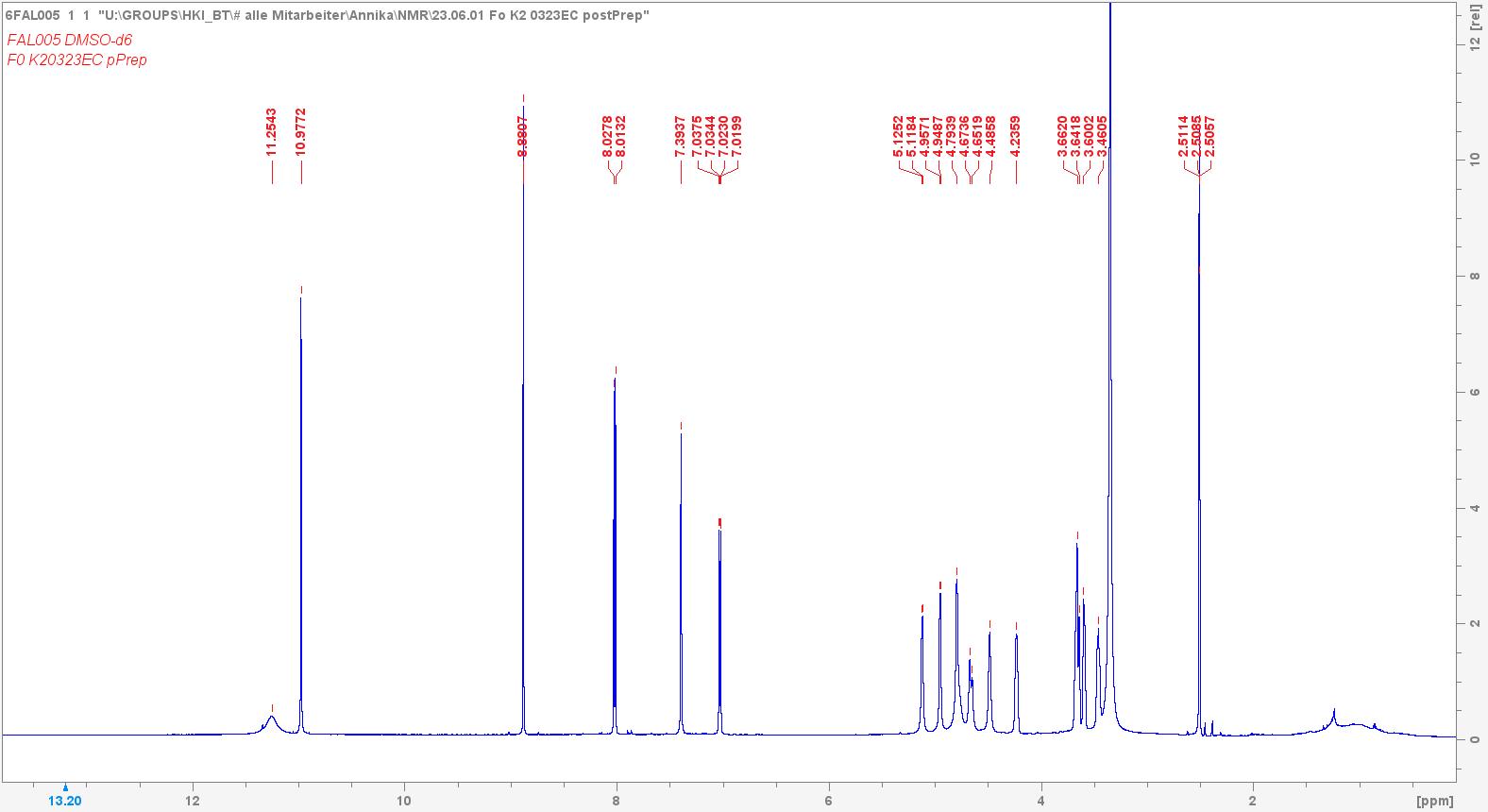


**Supplementary Figure 7: ^1^H NMR spectrum of purified F_O_, dissolved in DMSO-d^6^ (3.33 and 2.51/2.50 ppm).**
